# Circ-AFAP1 promote clear cell renal cell carcinoma growth and angiogenesis by the Circ-AFAP1/miR-374b-3p/VEGFA signaling axis

**DOI:** 10.1038/s41420-022-00865-1

**Published:** 2022-02-16

**Authors:** Yuxi Ou, Xiyu Dai, Xinan Chen, Yiling Chen, Siqi Wu, Quan Zhou, Chen Yang, Haowen Jiang

**Affiliations:** 1 Department of Urology, Huashan Hospital, Fudan University, Shanghai, China; 2 Fudan Institute of Urology, Huashan Hospital, Fudan University, Shanghai, China; 3 National Clinical Research Center for Aging and Medicine, Fudan University, Shanghai, China

**Keywords:** Renal cell carcinoma, miRNAs

## Abstract

Clear cell renal cell carcinoma (ccRCC) is one of the most common urogenital tumors with high mortality. Circular RNA (circRNA), as an emerging endogenous RNA, has been proved to play a crucial role in the clear cell renal cell carcinoma (ccRCC) progression. In this study, we obtained circAFAP1 upregulated in ccRCC by high-sequencing and verified by qRT-PCR in several renal cancer cell lines. In situ hybridization (ISH) assays and Kaplan–Meier plot showed a higher level of circAFAP1 was linked to shorter overall survival. Moreover, CCK8, colony formation, and EdU experiments showed circAFAP1 promoted ccRCC growth while tube formation displayed circAFAP1 contributed to ccRCC angiogenesis. We predicted the downstream miR-374b-3p and VEGFA by bioinformatic analysis and validated further by qRT-PCR, RNA pull-down, RIP, and dual-luciferase. Downregulation miR-374b-3p or overexpression VEGFA could restore proliferation, vascular formation after circAFAP1 silencing. Consistently with the results in vitro, silencing circAFAP1 suppressed ccRCC growth in vivo. In conclusion, the circAFAP1/miR-374b-3p/VEGFA axis played a critical role in the progression and development of ccRCC which might be novel biological marks and therapeutical targets.

## Introduction

Renal cell carcinoma (RCC) is one of the most common urogenital tumors all over the world and causes ~180,000 mortalities during 2020 [1]. RCC is composed of several histological subtypes, and about 70% of RCC patients are diagnosed exactly with clear cell RCC (ccRCC) [2]. Surgery excision of RCC serves as the main treatment, while tumor recurrence remains about 30% after radical nephrectomy which leads to metastasis-related mortality [3]. Furthermore, resistance to neoadjuvant RCC treatment as radiation therapy and chemotherapy would eventually lead to an unsatisfied overall survival of RCC which intrigues us for exploring novel therapeutic agents and biomarkers for diagnosis [4].

CircRNA was generated through non-classical back-splicing of mRNA to a circular configuration and possess more stability than its linear form [5, 6]. CircRNA could function potentially as a stable novel biomarker in diseases diagnosis and treatment since it was resistant to RNase R [7–9]. Competitive endogenous RNA mechanism is a well-recognized ncRNA regulatory mechanism of circRNA in regulating biological processes [5, 9]. CircRNA could directly bind with the miRNA as a sponge leading to post-transcriptional modulation of the downstream target [10].

Several previous studies identified circular RNA as closely correlated with the tumor progression of ccRCC. Circ-AKT3 suppressed RCC progression via regulating miR-296-3p/E-cadherin signals, while circTLK1 interact with miR-136-5p to promote the malignant phenotype of RCC [11, 12]. Thus, more uncovered circRNAs in RCC should be further characterized and investigated.

In this study, we firstly identified circAFAP1 as a promoter of RCC progression and had the potential of serving as a diagnostic biomarker. High expression of circAFAP1 was associated with unfavorable clinical outcomes. Also, circAFAP1 could promote the cell viability of RCC cells and vascular formation in RCC. Furthermore, we found circAFAP1 could modulate RCC progression by acting as the sponge of miR-374b-3p and upregulation of VEGFA. In conclusion, circAFAP1 has the potential to become a biomarker and therapeutic target in RCC surveillance and treatment.

## Result

### circAFAP1 is upregulated in ccRCC and high expression of circAFAP1 predicted an unfavorable prognosis

**Fig. 1:**
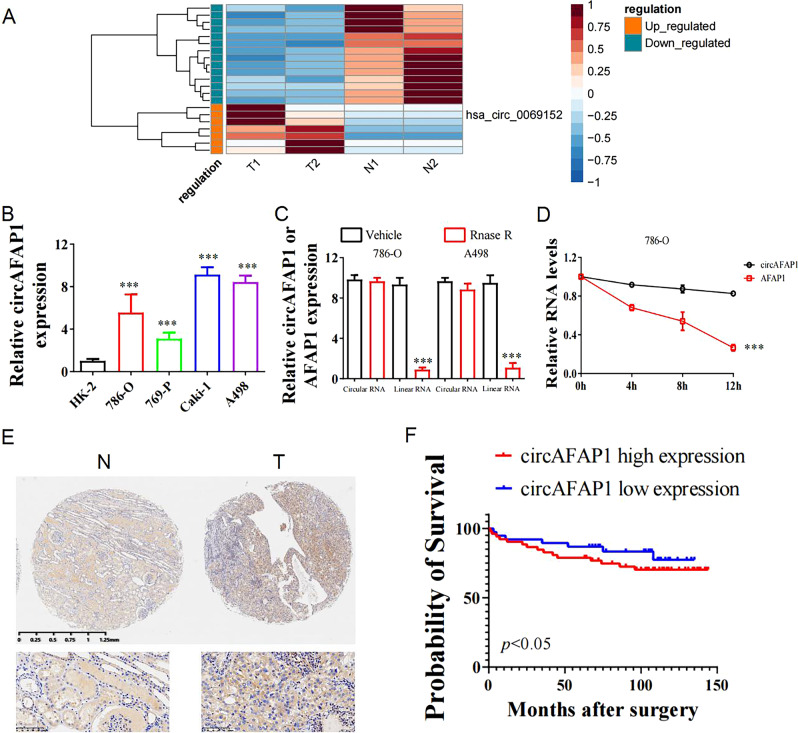
circAFAP1 is upregulated in ccRCC and high expression of circAFAP1 predicted an unfavorable prognosis. **A** Cluster heatmap for 21 deregulated circRNAs in two paired ccRCC and pericarcinomatous tissues (filtered by *P* < 0.05 and |FC (fold change)| ≥ 2). Columns represent tissues while rows for differentially expressed circRNAs, with circAFAP1 identified on the right of its rows. **B** qRT-PCR was analyzed for the expression of circAFAP1 in normal renal tubular epithelial cells line HK-2 and ccRCC cell lines 786-O, 786-P, Caki-1, and A498. Data were shown as the means ± SD. ***represents *p* < 0.001. **C** qRT-PCR was analyzed for expression of circAFAP1 and linear mRNA-AFAP1 in 786-O and A498 cells treated with or without RNase. Data were shown as the means ± SD. ***represents *p* < 0.001. **D** qRT-PCR detected the residual level of circAFAP1 and linear mRNA-AFAP1 in 786-O cells treated with Actinomycin D at the specific time point. Data were shown as the means ± SD. ***represents *p* < 0.001. **E** Tissue microarray data in 82 paired ccRCC and pericarcinomatous tissues stained by immunohistochemistry revealed circAFAP1 is upregulated in ccRCC tissue (Right panel) compared to pericarcinomatous tissues (Left panel). **F** Survival curves of 82 ccRCC patients with high or low expression, according to the immunohistochemical staining level and using the median value as the grouping criterion. The follow-up time spans 150 months after surgery.

In order to explore the biological correlations between circRNA expression and ccRCC progression, we extracted and analyzed total RNA from two paired ccRCC and pericarcinomatous tissues. We posted seven significantly upregulated and 14 significantly downregulated circRNAs identified by differential gene expression analysis between normal and tumor tissues. We intended to select the upregulated circRNAs in RCC to further discover their biological role which upregulated circRNA has the potential to be a biomarker of RCC while the overall expression of circRNA in cancer remains low. The differentially expressed circRNAs could possess the property to relate with the tumorigenesis and tumor progression of ccRCC. We highlighted the potential of circAFAP1 because of its significant upregulation in ccRCC (Fig. 1A). Next, we performed qRT-PCR to figure out circAFAP1 expression in different cell lines composed of tumor cells (786-O, 786-P, Caki-1, and A498) and immortalized normal renal cells (HK-2). All of these four renal carcinoma cell lines were significantly highly expressed circAFAP1 compared to normal renal tubular epithelial cells line HK-2 (Fig. 1B). As we expected, circAFAP1 showed greater resistance to RNase A digestion (Fig. 1C) and greater stabilization under Actinomycin D treatment than its linear form (Fig. 1D), which indicates circAFAP1 transcript is of circular form. Furthermore, we verified circAFAP1 upregulated in 82 ccRCC tissues than their paired pericarcinomatous tissues by in situ hybridization (ISH) on tissue microarray (Fig. 1E). According to the ISH staining level, we divided these patients into a high or low expression group using the median value as the grouping criterion. The Kaplan–Meier plot displayed the high expression group having a worse prognosis than the low expression group (Fig. 1F), which suggested circAFAP1 could serve as a marker to evaluate the prognosis of ccRCC. It interested us to explore the underlying mechanism of this discovery and whether circAFAP1 could make a difference to the ccRCC oncogenesis and progression.

### circAFAP1 promotes proliferation and vascular formation in ccRCC cells

**Fig. 2:**
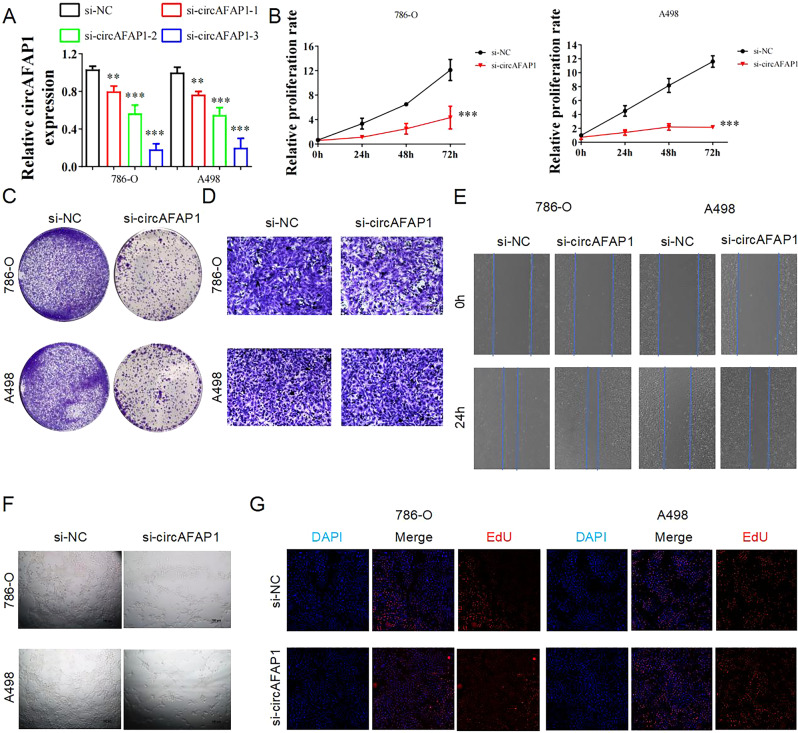
circAFAP1 promotes proliferation and vascular formation in ccRCC cells. **A** qRT-PCR for expression of circAFAP1 revealed that all the three siRNAs can reduce the expression in 786-O and A498 cells, while si-circAFAP1-3 displayed the most powerful silencing efficiency. ***represents *p* < 0.001. **represents *p* < 0.01. **B**, **C** CCK8 proliferation assays (**B**) and colony formation assays (**C**) displayed that the 786-O and A498 cells transfected with si-circAFAP1 have abrogated cell proliferation ability. ***represents *p* < 0.001. **D** Transwell assays were applied to discover the invasion ability after si-circAFAP1 transfection in 786-O and A498 cells. **E** Wound healing was applied to discover the migration ability after si-circAFAP1 transfection in 786-O and A498 cells. **F** Tube formation assay was applied to further validate the vascular promotion ability after adding the supernatant of si-circAFAP1 transfection of 786-O and A498 cells in HUVEC cells. **G** EdU assays were applied to further verify si-circAFAP1 inhibited the proliferation ability of 786-O and A498 cells.

To explore what role the circAFAP1 plays in ccRCC oncogenesis and progression, we artificially synthesized three siRNAs targeting its back-splicing region. All the three siRNAs could reduce the expression, detected by qRT-PCR, in 786-O and A498 cells, while si-circAFAP1-3 displayed the most powerful silencing efficiency (Fig. 2A). Then, the CCK8 assays (Fig. 2B) and colony formation assays (Fig. 2C) revealed that the proliferation ability of 786-O and A498 cells were inhibited after knockdown of circAFAP1. However, transwell assays showed that circAFAP1 had no obvious effect on the invasion ability of ccRCC cells (Fig. 2D) while wound-healing assay discovered no significant change in migration ability after knockdown of circAFAP1 in ccRCC cells (Fig. 2E). Since enhanced proliferation ability of ccRCC cells may contribute to more angiogenesis in ccRCC through the secretion of cytokines, we further analyzed the vascular formation ability of HUVEC cells while cultured with the supernatant of si-circAFAP1 ccRCC cells. Tube formation assay demonstrated circAFAP1 can promote angiogenesis (Fig. 2F) which indicated vascular promotion ability may contribute to the underlying mechanism of circAFAP1 to promote ccRCC oncogenesis and progression. EdU assays were applied to further verify that silencing circAFAP1 suppressed ccRCC cells growth (Fig. 2G). Taken together, we prove that circAFAP1 promotes proliferation and vascular formation in ccRCC cells.

### circAFAP1 serves as a miR-374b-3p sponge to regulate VEGFA expression in ccRCC cells

**Fig. 3:**
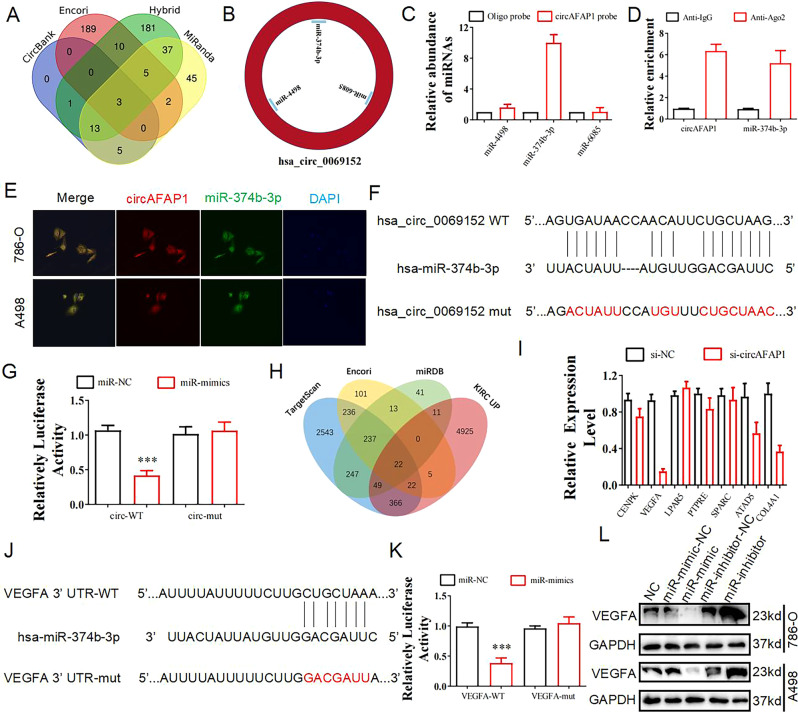
circAFAP1 acts as a miR-374b-3p miRNA sponge in ccRCC cells and miR-374b-3p serves as an mRNA suppressor to regulate VEGFA expression. **A** Venn diagram predicted three potential targeted miRNAs binding with circAFAP1 from circbank, Encori, Hybrid, and MiRanda database. **B** miR-4498, miR-6085, miR-374b-3p were acquired from four before-mentioned software programs. **C** RNA pulldown assay supplied with qRT-PCR displayed circAFAP1 was bound with three miRNAs while the interaction between circAFAP1 with miR-374b-3p was strongest. Error bars indicate SD. **D** RIP experiments were performed in 786-O cells against IgG or Ago2. The results analyzed by qRT-PCR supported the correlation between circAFAP1 with miR-374b-3p. **E** FISH showed the subcellular co-localization between circAFAP1(red) and miR-374b-3p(green). DAPI (blue) was used to stain the nuclear. **F** Dual-luciferase reporter assays verified the exact binding site where circAFAP1 interaction with miR-374b-3p and mutated circAFAP1 sequence was shown as the last row. **G** The luciferase activity was determined 2 days after transfected with miRNA mimics normal control (miR-NC)/miR-374b-3p mimics (miR-mimics) in 786-O cells. ***represents *p* < 0.001. **H** Venn diagram presented potential targeted mRNAs regulated by miR-374b-3p from Targetscan, miRDB, Encori, and TCGA database. **I** qRT-PCR showed seven mRNAs were differentially expressed in 786-O cells with or without transfecting si-circAFAP1 while the most significant difference between these two kinds of cells was observed in VEGFA. **J** Dual-luciferase reporter assays verified the exact binding site where VFGFA mRNA interaction with miR-374b-3p and mutated VFGFA mRNA sequence was shown in the last row. **K** The luciferase activities were determined 2 days after transfecting miRNA mimics negative control (miR-NC)/miR-374b-3p mimics (miR-mimics) in 786-O cells. ***represents *p* < 0.001. **L** Western blot displayed the VEGFA levels among 786-O and A498 cells, both of which were transfected with miR-NC, miR-mimic, miR-inhibitor-NC, or miR-inhibitor respectively. GAPDH was used as the reference gene.

Several researches have identified the interaction between circRNA and miRNA to regulate downstream genes expression [6, 13–15]. We screened and obtained three miRNAs (Fig. 3A), miR-4498, miR-6085, and miR-374b-3p, as the possible downstream miRNAs of circAFAP1 predicted by circbank, Encori, Hybrid, and MiRanda database (Fig. 3B). To explore the accurate downstream miRNA of circAFAP1, RNA pulldown assays were carried out and showed that interaction between circAFAP1 with miR-374b-3p was strongest among three miRNAs (Fig. 3C). Next, we conducted Ago2 RNA immunoprecipitation (RIP) experiments to verify higher circAFAP1 and miR-374b-3p levels in anti-Ago2 RIP compared to anti-immunoglobulin G (IgG) RIP (Fig. 3D). Consistently, fluorescence in situ hybridization (FISH) showed the fitting subcellular co-localization between circAFAP1 and miR-374b-3p (Fig. 3E). Furthermore, we designed two dual-luciferase reporter vectors, containing wild-type circAFAP1 sequence and mutant circAFAP1 sequence respectively, then cloned and co-transfected with miR-374b-3p mimics or miR-NC into 786-O cells (Fig. 3F). The luciferase activities showed co-transfection of wild-type reporter vectors and miR-374b-3p mimics reduced the luciferase activity significantly while co-transfection of mutant reporter vectors and miR-374b-3p mimics made no difference to luciferase activity. In conclusion, miR-374b-3p is exactly the downstream target of circAFAP1 (Fig. 3G).

Next, we dedicated ourselves to further identifying the downstream target of miR-374b-3p. We found 22 potential downstream target mRNA of miR-374b-3p from Targetscan, miRDB, Encori, and TCGA database (Fig. 3H). We further selected seven downstream mRNAs in consideration of their already discovered role in carcinogenesis. qRT-PCR showed seven mRNAs were differentially expressed in 786-O cells with or without transfecting si-circAFAP1 while the most significant difference was observed in VEGFA mRNA (Fig. 3I). Since silencing circAFAP1 significantly hampers the vascular formation ability in ccRCC cells, we speculate circAFAP1 may regulate VEGFA expression to modulate angiogenesis [16]. We performed a dual-luciferase assay and the luciferase activity of cells transfected wild-type VEGFA mRNA sequence significantly diminished after miR-374b-3p mimics transfection while luciferase activity was not changed in cells transfected with mutated VEGFA mRNA sequence (Fig. 3J, K). Western blot illustrated that miR-374b-3p could downregulate the expression of VEGFA while miR-374-3p inhibition upregulated the expression of VEGFA (Fig. 3L).

### Downregulation miR-374b-3p or overexpression VEGFA could restore proliferation, vascular formation, and p-erk expression after circAFAP1 silencing

**Fig. 4:**
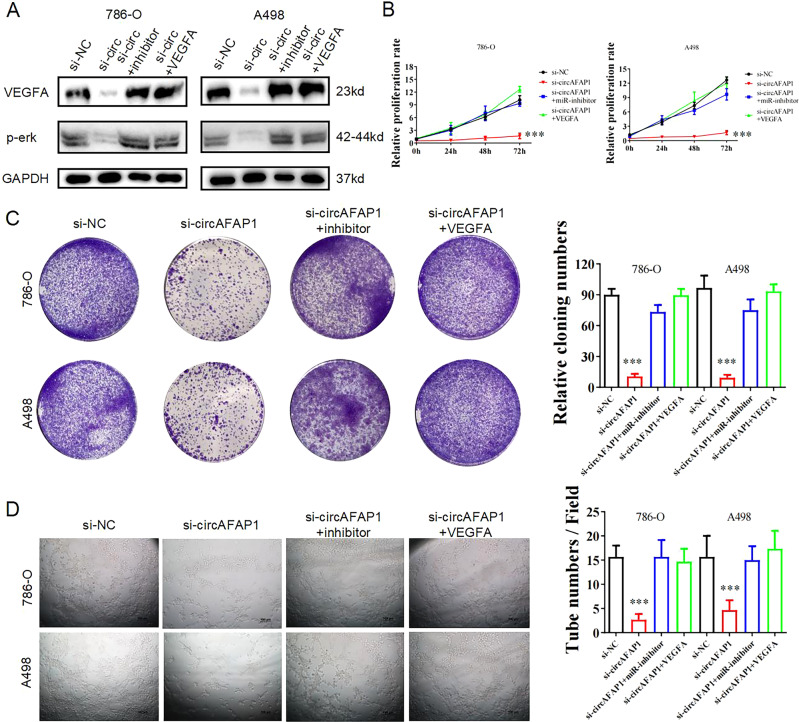
Downregulation miR-374b-3p or overexpression VEGFA could restore proliferation and vascular formation in ccRCC cells after circAFAP1 silencing. **A** Western blot displayed the VEGFA and p-erk levels in 786-O and A498 cells, both of which were transfected of scrambled siRNA (si-NC), si-circAFAP1 (si-circ), si-circAFAP1+miRNA inhibitor (si-circ +inhibitor), or si-circAFAP1+VEGFA overexpression plasmid (si-circ + VEGFA) respectively. GAPDH was used as the control gene. **B**, **C** CCK8 proliferation assays (**B**) and colony formation assays (**C**) were carried out to evaluate the proliferation ability. ***represents *p* < 0.001. **D** Tube formation assay was applied to measure the vascular formation ability after adding the supernatant of the relative ccRCC cells into HUVEC cells. ***represents *p* < 0.001.

In order to explore the accurate relationships between circAFAP1, miR-374b-3p, and VEGFA, we created kinds of transfected 786-0 cells in which miR-374b-3p was silenced or VEGFA was upregulated. In previous studies, VEGFA could activate Ras/ERK signaling to promote bladder cancer cell proliferation, differentiation, and migration. Hence, we performed western blot experiments to detect the expression of VEGFA and p-erk in transfected 786-0 cells. The results showed the expression of VEGFA and p-erk were suppressed after silencing circAFAP1, but miR-374b-3p suppression or VEGFA upregulation restored the situation (Fig. 4A). Subsequently, circAFAP1 knockdown reduced the proliferation ability of ccRCC cell lines verified by CCK8 assays and colony formation assays whereas co-transfected miR-374b-3p inhibitor counteracted this effect to a large extent and restored completely the reduction after VEGFA overexpression (Fig. 4B, C). Moreover, based on the suppression effects on the vascular promotion ability that transfected si-circAFAP1 displayed in tube formation assay, downregulation miR-374b-3p or overexpression VEGFA recovered the degree of angiogenesis (Fig. 4D). Therefore, we confirmed miR-374b-3p downregulation or VEGFA overexpression could restore proliferation, vascular formation, and p-erk expression after circAFAP1 silencing.

### Silencing circAFAP1 suppressed ccRCC growth in vivo

**Fig. 5:**
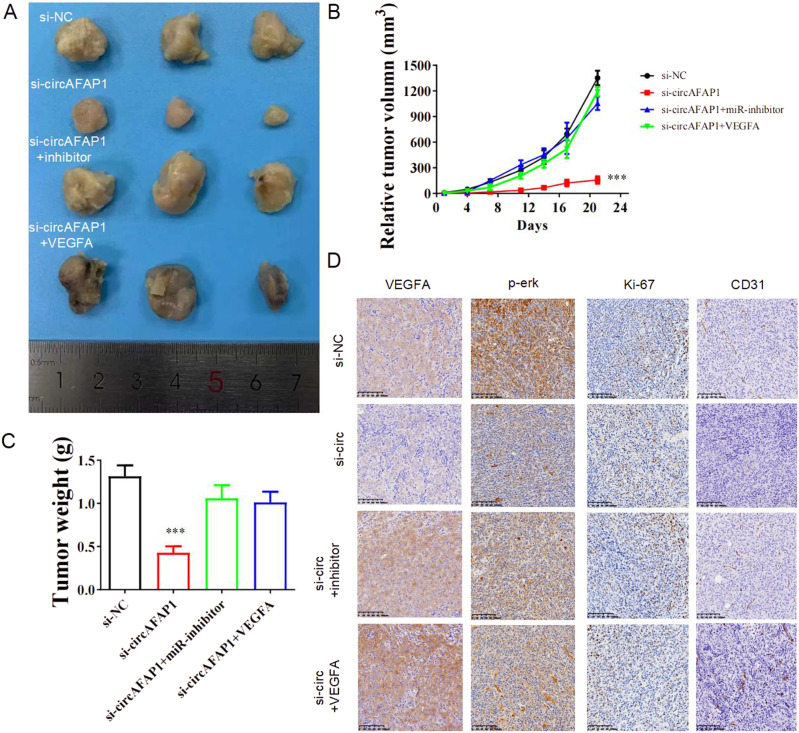
Silencing circAFAP1 suppressed ccRCC growth in vivo. **A** Transfected 786-O cells were subcutaneously injected and sacrificed 21 days after injection. **B**, **C** Each of tumor volumes (**B**) and weights (**C**) was measured individually and displayed. ***represents *p* < 0.001. **D** Immunohistochemical staining displayed the expression of VEGFA, p-erk, ki-67, and CD31 in the tumor tissue of nude mice.

We study further the mechanism underlying the difference which circAFAP1 makes to ccRCC in vivo. 786-O cells transfected with scrambled RNA (si-NC), si-circAFAP1-3 (si-circ), si-circAFAP1-3 accompanied with miR-374b-3p inhibitor (si-circ + inhibitor), and si-circAFAP1-3 coupled with VEGFA overexpression vector (si-circ + VEGFA) were subcutaneously injected and the tumor tissues taken out and measured 21 days after injection (Fig. 5A). The same as the results in vitro, knockdown circAFAP1 reduced markedly volumes and weights of ccRCC tissues, while miR-374b-3p downregulation or VEGFA overexpression rescued the suppression effects (Fig. 5B, C). In addition, immunohistochemical staining revealed knockdown circAFAP1 decreased the expression of VEGFA, p-erk, ki-6,7, and blood vessel marker CD31 in tumor tissues which were restored by miR-374b-3p suppression or VEGFA upregulation (Fig. 5D).

## Discussion

Several circRNAs have been discovered in the past decades [11, 17, 18]. In this work, we identified a novel circRNA highly expressed in ccRCC named circAFAP1, which was significantly correlated with clinical stages and prognosis. Upregulation of circAFAP1 could promote ccRCC cell proliferation and angiogenesis in vitro and in vivo. Mechanistically, circAFAP1 functioned as a ceRNA of miR-374b-3p to diminish the inhibitory effect of VEGFA, which activated the Ras/ERK pathway in the progression of ccRCC.

Growth factor vascular endothelial growth factor A (VEGFA) mainly functions as an inducer of angiogenesis during physiologic and pathologic progress which is critical to ccRCC initiation and malignant phenotype [16, 19–21]. Sustainable growth and distant metastasis of cancer cells rely on angiogenesis [22]. We further validated the activation of the Ras/ERK signaling pathway after VEGFA overexpression.

Formed through back-splicing, circRNA serves as miRNA sponges and competitively binds to miRNAs directly, which could suppress the activity of miRNAs targeting mRNA [6, 17]. Furthermore, we demonstrated circAFAP1 functioned as a miRNA sponge for miR-374b-3p in ccRCC. We analyzed and took the intersection of the predicted target of circAFAP1 in circBank, Encori, RNA Hybrid, and MiRanda. miR-4498, miR-6085, and miR-374b-3p were selected and RNA pulldown assay identified the directed interaction between circAFAP1 and miR-374b-3p.

circAFAP1 could bind directly to miR-374b-3p in an AGO2-dependent manner. We analyzed and took the intersection of the predicted target of miR-374b-3p between Targetscan, Encori, miRDB, and upregulated genes in TCGA-KIRC and found 22 potential genes. We then review the function of these genes and perform RT-qPCR of these genes in si-circAFAP1 RCC cells. We verified VEGFA as our downstream target because VEGFA is important to RCC progression and remains downregulated in ccRCC cells.

Also, bioinformatics analysis and dual-luciferase reporter assay indicated 3′ UTR of circAFAP1 and VEGFA shared complementary miR-374b-3p binding sequence. Rescue assay further indicated circAFAP1 could regulate VEGFA expression in ccRCC via antagonizing miR-374b-3p.

In conclusion, circAFAP1 was found to be a prognosis-related oncogene in ccRCC. circAFAP1 is a miRNA sponge for miR-374b-3p targeting VEGFA to promote ccRCC growth and angiogenesis through the activation of the Ras/ERK signaling pathway. circAFAP1/miR-374b-3p/VEGFA axis played a critical role in the progression and development of ccRCC which might be novel biological marks and therapeutical targets.

## Materials and methods

### Patient samples and circRNA sequencing

A tissue microarray (TMA) was collected from ccRCC and adjacent normal tissue of 82 ccRCC patients who received partial or radical cystectomy in Huashan Hospital, Fudan University between 2008 and 2014. All tissues were deposited in liquid nitrogen or 4% PFA for future experiments and examined by two pathologists to confirm the diagnoses histologically. Written informed consent was acquired from each participant. The project was approved by the Board and Ethics Committee of Huashan Hospital, Fudan University (Approval number KY2011-009).

Total RNA was extracted with the treatment of RNase R and then subjected to high throughput sequencing. Differential gene expression analysis of circRNA was performed. The differentially expressed circRNAs were defined as the one with *P* < 0.05 and |log_2_FC | >2.

### Quantitative reverse transcriptase PCR (qRT-PCR)

Total RNA was first isolated from tissues or cells with TRIzol Reagent (Thermo Fisher Scientific, Invitrogen) by the manufacturer’s instructions.

### Cell transfection and vector construction

786-O and A498 cells were obtained from ATCC and authenticated through STR profiling and tested for mycoplasma. Lipofectamine 2000 (Invitrogen, USA) was used to transfect shRNA, siRNA, and miRNA mimics and inhibitors (GenePharma, Shanghai, China) into cells. All primers and oligo sequences are listed in supplementary data.

### RNA in situ hybridization (ISH) and immunohistochemistry (IHC)

CircAFAP1 expression in ccRCC was determined via biotin-labeled probes in a TMA as described previously [23]. Tumor samples were embedded and stained with protein markers [24]. Ki-67 (27309-1-AP) and CD31 (11265-1-AP) were selected for the IHC assay.

### RNA fluorescence in situ hybridization (FISH)

Specific probes to circAFAP1 and probes against miR-374b-3p were prepared (Geneseed Biotech, Guangzhou, China) and the FISH assay was performed as previously described.

### RIP assay

RIP assay was performed with the Magnetic RIP RNA-binding protein immunoprecipitation kit (Millipore) [24].

### RNA pulldown

Probe coated beads were constructed with incubation of biotin-labeled circAFAP1 or oligo probes with Streptavidin-coupled magnetic beads (Life Technologies, USA), and then for RNA pulldown analysis [24].

### Western blotting analysis

Protein was extracted from cells lysed in ice-cold RIPA lysis buffer and separated via SDS-PAGE to analyze relative protein expression as in our previous work. VEGFA (CST, 65373), p-ERK (CST, 4370), and GAPDH (Proteintech, 60004-1-lg) were selected for WB assay.

### Cell counting kit-8 assay

Cell Counting Kit-8 assay (Sigma-Aldrich) was applied for cell proliferation assessment. Cells were seeded into 96-well plates and cell viability was assessed at 0, 24, 48, and 72 h after cell seeding.

### Colony formation assay

Cells were seeded into six-well plates at 500 cells per well. The colonies were then fixed with 4% PFA and stained with crystal violet 8 days after culturing in complete DMEM.

### Tube formation

HUVEC was seeded in 12-well plates supplemented with Matrigel and then seeded under ccRCC cell-conditioned media. After incubated for 12 h, tube formation of HUVEC was then imaged.

### Transwell assay

Cell invasion were assessed through Transwell chambers (Costar) according to the manufacturer’s procedure.

### Wound-healing assay

About 30,000 ccRCC cells per well were seeded into a six-well plate and a straight scratch was made. Cells proliferation was inhibited with 5 ug/ml mitomycin C in complete DMEM. The wounds were evaluated 24 h after scratching.

### Luciferase reporter assay

Wild-type and mutated cDNA fragments of circAFAP1 and VEGFA 3′-UTR were cloned and recombined into psiCHECK-2 (Promega, Madison, WI, USA) to perform a dual-luciferase reporter assay as previously described [25].

### Animal study

A total of 1 × 10^7^ 786-O transfected with si-NC, si-circAFAP1, si-circAFAP1 + miR-374b-3p, or si-circAFAP1 + VEGFA were injected subcutaneously into 4-week-nude mice (five per group) and monitored every 5 days. Mice were sacrificed after 21 days and tumors were excised and weighed. The animal experiment was performed according to the Ethics Committee of Fudan University (Approval number 2021JS-173). The animals are selected and allocated to each treatment group according to their weight before treatment.

### Bioinformatics analysis

CircBank, Encori, Circinteractome, MiRanda, TargetScan, miRWalk, and TCGA was applied to discover the interaction of circRNA/miRNA/mRNA. Patients in TCGA-KIRC was divided into high/low groups based on the expression of VEGFA.

## Supplementary information

Primers and probes sequences

## Data Availability

The analysed data sets generated during the study are available from the corresponding author on reasonable request.
