# Supplementary material for: Circ-AFAP1 promote clear cell renal cell carcinoma growth and angiogenesis by the Circ-AFAP1/miR-374b-3p/VEGFA signaling axis
Source: Cell Death Discov. 2022 Feb 16;8:68. doi: 10.1038/s41420-022-00865-1 (PMC8850424; doi:10.1038/s41420-022-00865-1)
Supplement: Supplementary file 1 — Primers and probes sequences [file 41420_2022_865_MOESM1_ESM.doc]

**RT-qPCR primers:**

GAPDH Forward 5-CAAGGCTGAGAACGGGAAG-3

GAPDH Reverse 5-TGAAGACGCCAGTGGACTC-3

AFAP1 Forward 5-CTCCTCCGGCTTTACACCAAA-3

AFAP1 Reverse 5-GGCAAGCTGTTTCCGTTCTG-3

circAFAP1 Forward 5-GTCCAGGAGTTCAAGAAAGA-3

circAFAP1 Reverse 5-GCTCAGAAGCAGGAGACC-3

CENPK Forward 5-AACACTCACCGATTCAAATGCT-3

CENPK Reverse 5-CAGTCAAGGGAATTGTTTCAGGT-3

VEGFA Forward 5-AGGGCAGAATCATCACGAAGT-3

VEGFA Reverse 5-AGGGTCTCGATTGGATGGCA-3

LPAR5 Forward 5-ACCTGGACATGATGTTTGCCA-3

LPAR5 Reverse 5-GAGACCAGTCGCCAATACCA-3

PTPRE Forward 5-CAGCACCAGCGACAAGAAGAT-3

PTPRE Reverse 5-CCACGGGGATGGGAAAATACTT-3

SPARC Forward 5-TTCTCGGCGCTCACGTTTTT-3

SPARC Reverse 5-GTTGAAATTCACAACGGGAAAGG-3

ATAD5 Forward 5-GTGAAGGACTGCGAGATTGAG-3

ATAD5 Reverse 5-TGTCTCTAGTCTTCCCTAGTGGT-3

COL4A1 Forward 5-GGACTACCTGGAACAAAAGGG-3

COL4A1 Reverse 5-GCCAAGTATCTCACCTGGATCA-3

miR-4498 5-ACACTCCAGCTGGGTGGGCTGGCAGGGCAA-3

miR-374b-3p 5-ACACTCCAGCTGGGCTTAGCAGGTTGTATT-3

miR-6085 5-ACACTCCAGCTGGGAAGGGGCTGGGGG-3

miRNA reverse primer 5-TGGTGTCGTGGAGTCG-3

U6 Forward 5-CTCGCTTCGGCAGCACA-3

U6 Reverse 5-AACGCTTCACGAATTTGCGT-3

**Probes:**

circAFAP1-Cy3: 5-TTAACTCTTCCATTGCAGCCAGGGCTGAGG-3

circAFAP1-Bio: 5-TGGCATTGAGATCATCTGGCATGCCATTCT-3

miR-374b-3p-Dig: 5-AATGATAATACAACCTGCTAAG-3
